# Social Determinants of Health and Depression: A Preliminary Investigation from Rural China

**DOI:** 10.1371/journal.pone.0030553

**Published:** 2012-01-19

**Authors:** Yuan Liang, Yan-Hong Gong, Xiao-Piao Wen, Chao-Ping Guan, Ming-Chuan Li, Ping Yin, Zhi-Qing Wang

**Affiliations:** 1 Department of Social Medicine and Health Management, School of Public Health, Tongji Medical College, Huazhong University of Science and Technology, Wuhan, China; 2 Department of Epidemiology and Biostatistics, School of Public Health, Tongji Medical College, Huazhong University of Science and Technology, Wuhan, China; 3 Department of Clinical Epidemiology, the Suicide Research and Training Center of World Health Organization & Beijing Suicide Research and Prevention Center, Beijing Hui Long Guan Hospital, Beijing, China; Federal University of Rio de Janeiro, Brazil

## Abstract

**Background:**

In the last several years, research related to social determinants of health (SDH) has begun to resonate in the medical, behavioral, social and political sciences arena. The aim of the present study was to explore the relationship between SDH and depression, and to provide new evidences and clues for depression control and prevention.

**Methodology/Principal Findings:**

This research was a cross-sectional survey executed door to door from October 2006 to April 2008, with a sample of 3,738 individuals aged 18 and older in rural China. The three variables of SDH were socioeconomic status (years of schooling and self-reported economic status of family), social cohesion and negative life events. Demographic variables and self-perceived physical health were taken as potential confounders. The cross-table analysis showed that variations in levels of depression were associated with variations in SDH, and logistic regression analysis confirmed the association even after adjusting for potential confounding variables.

**Conclusions:**

Although there were some limitations, the current study provides initial evidence of the importance of SDH in depression. Findings indicate that social inequity and the role of policy action emphasized by SDH should be considered high priorities when addressing the issue of depression. In addition, cell-to-society and pill-to-policy approaches should be encouraged in the future.

## Introduction

During the past few decades, the depth and breadth of our understanding of health issues has greatly increased because of the bio-psycho-social medical model [1]–[3]. Important to this understanding is the concept of Social Determinants of Health, SDH [4]–[6]. According to the Commission on Social Determinants of Health (CSDH) of the World Health Organization (WHO), set up in 2005 by Dr. Lee Jong-wook, the former Director-General of WHO, SDH focus on the “causes of the causes”-“the fundamental structures of social hierarchy and the socially determined conditions these structures create in which people grow, live, work and age” [7]–[8]. SDH are primarily responsible for health inequities—the unfair and avoidable differences in health status. Variations in social factors are determinate influences on variations in health among people, and the health inequity is the result of social inequity, a problem needing policy responses and action [4]–[8]. In addition, reports from the Institute of Medicine and the Robert Wood Johnson Foundation's Commission to Build a Healthier America have stated that social determinants not only contribute to risk and resilience in health, but are also important considerations for interventions beyond the individual to macro levels including neighborhoods, communities, and public policy [9]–[12].

In recent years, findings and bold recommendations related to SDH are evident in the medical, behavioral, social and political sciences fields [13]–[15]. SDH can address communicable and non-communicable diseases, as well as physical and mental health. A few researchers have started to pay attention to SDH in related to mental health issues, including depression [16]–[20]. Mental health can only be understood by considering the biological, social, cultural, economic and personal contexts of their lives [21]–[23]. These perspectives are testimony to the breadth of mental health and how the field has advanced in the last two decades. Recently, although researchers have suggested that depression research needs to broaden its focus and address SDH, little empirical research has been reported on SDH and mental health [16]–[7,24].

In addition, researchers have often cited social factors related to health over the past few decades. Although these factors are very similar to SDH in form, there is an important difference. The emphasis of SDH is on the relationship between variations in health (health inequity) and in social factors (social inequity). If social factors are risk factors for poor health, then social inequality represents the “causes of the causes” focused on by the WHO-CSDH. Policy action has been advocated by the WHO-CSDH as social treatment targeted at social inequality. Social inequality (not simplified as social factors) has a significant impact on in physical and mental health. Public policy, as a tool for dealing with social inequity, has an important role in SDH [7], [8], [14], [25].Various models of public policy provide paths by which social inequity barriers can be overcome, including the policies to reduce poverty and racial segregation as well as promote education, healthier homes, neighborhoods, schools and workplaces. Furthermore, these issues should be addressed not only by those within the health sector and by psychiatrists, but also by intersectoral policy action and government [7], [8], [14], [24].

In short, there is a relative dearth of past research on the SDH and mental health. What little research there is suggests that SDH may be influential, at least upon mental health, but there are still a number of important gaps in knowledge. To address these gaps, the current study offers a preliminary investigation into the relationship between three key SDH (socioeconomic status, social cohesion and negative life events) and depression using community-based data in rural China. We hypothesized that variations in levels of depression would be associated with variations in socioeconomic status, social cohesion and negative life events. We want to provide new evidences and clue for theoretical innovations in controlling depression as well as for prevention in the future.

## Methods

### Participants

This was a cross-sectional survey executed door to door and face to face. Data were collected from October 2006 to April 2008. The study population was residents 18 years of age and older from rural areas in Tuanfeng, Hubei Province, in central China. We used a stratified random sample of the entire group. From the 10 townships in Tuanfeng, five townships were randomly selected, and then 22 villages were randomly selected from the 94 villages of those townships. All residents of the 22 villages were included. The total sample comprised 6,274 individuals. Among them, 1,856 (29.58%) were not at home at the time of the survey visit (e.g., working in the fields, visiting relatives, visiting the doctor or hospital), 377 (6.01%) declined to participate and 271 (4.32%) had a physical or mental disorder that was a barrier to participation. Thus, 3,770 (60.09%) of the 6,274 sampled were interviewed by trained interviewers. Some questionnaires were discarded because of missing data. Finally, 3,738 usable questionnaires remained, with an overall response rate of 59.58%. All residents provided written informed consent. We took measures to ensure valid results, which included training interviewers before the survey and supervision during the survey procedure.

### Measures

#### Depression

We used the Beck Depression Inventory-II [26], a 21-item scale designed to measure symptoms of depression. Respondents are asked to rate for the previous week the severity of each item on a scale of 0 to 3. Total scores range from 0 to 63. The BDI-II manual recommends that an index score<14 suggests no depression, 14 to 19 suggests mild depression, and ≥20 suggests moderate or severe depression. In this study, depression cut off scores were consistent with the BDI-II manual. The BDI-II has high internal consistency (α = 0.892).

#### Social determinants of health

Based on existing research [7]–[8], [20], we used three key SDH: socioeconomic status, social cohesion and negative life events. Socioeconomic status included two indicators: years of schooling and self-reported economic status of the family, in general, in the previous year. Categories for years of schooling were as follows: above average (7 years and above), average (1–6 years) and below average (0 years). Economic status of the family was self-reported as good, average or poor. Social cohesion was assessed from responses to two questions: (1) In the previous year, how often did you ask someone for help when you had problems? (Never = 1; Seldom = 2; Sometimes = 3; Often = 4), and (2) At any time in the past, did the following individuals or organizations give you help when you had problems? (spouse or lover; parents, brothers, sisters or children; other relatives; people outside the family; organization or schools with whom you are affiliated; government, party or trade unions; religious or non-governmental organizations; other organizations) (no = 0; yes = 1). Negative life events were assessed using a 12-item scale (serious illness in oneself, serious illness in the family, financial difficulties, conflict with spouse, conflict with other family members, conflict with people in the village, conflict between family members, infertility issues, problems at work or school, problems in an intimate relationship, abuse and other events) [27]. For each life event that occurred in the last year, or that occurred earlier but continued to have a psychological effect during the past 12 months, the respondent indicated when the life event occurred, its effect (positive or negative) and the length of time over the last year that the psychological effect lasted. We used the sum of the number of life events with a negative effect as a measure of negative life events.

#### Potential confounding variables

Variables that may influence symptoms of depression were included as controls. Including these variables in the models statistically removed their effect on the dependent variable. Demographic characteristics that are frequently associated with symptoms of depression, such as gender, age and marital status (never married, married, remarried, cohabitating or other) were used as potential confounding variables. In addition, some researches indicated that the role of physical health on depression is significant [28]–[29]. Thus, in our research, self-perceived physical health in the previous month was used as a potential confounding variable, with the following response choices: good, fair and poor.

### Statistical analysis

All analyses were performed using SPSS, version 12.0 (SPSS Inc., Chicago, IL, USA). Descriptive analysis was carried out for demographics data and SDH. The association between variations in depression symptoms and variations in SDH was shown with cross-tabulation, and Pearson's x^2^ tests were used to examine the statistical significance of deviations between both. Logistic regression analysis was used to analyze risk factors (demographic variables, self-perceived physical health and SDH as independent variables and depression status as the dependent variable). First, crude odds ratios (ORs) and 95% confidence intervals (CIs) for each variable were calculated using univariable logistic regression. Second, the independence of any association was examined by controlling for significant potential confounding variables such as age, marital status and self-perceived physical health status. Then, through a stepwise logistic analysis, with a significance level of 0.05 to enter and to stay in the model, final multivariable logistic regression model including all significant variables was conducted. For all comparisons, differences were tested using the two-tailed test and *p*-value less than 0.05 was considered statistically significant.

## Results

The descriptive statistics for the primary variables in the study are provided in Table 1. The age of the sample ranged from 18 to 92 years old (M = 53.09; SD = 14.66). The majority of our sample was female (59.98%) because many rural men work in the fields during the day and do not go home until evening. Therefore, our investigators could not meet with them in the daytime. With respect to level of education, 1395 participants (37.32%) were illiterate, which was expected for rural central China. According to self-reports, the prevalence of moderate and severe depression was 9.39% (351/3738) among the study population, and the prevalence of mild depression was 10.70% (400/3738).

**Table 1:** Descriptive statistics of the total study population (N = 3738).

Variables	N	%
**Dependent variable: symptoms of depression**		
Moderate and severe depression (20 points and above)	351	9.39
Mild depression (14–19 points)	400	10.70
No depression (0–13 points)	2987	79.91
**The variables of Social Determinants of Health**		
Self-reported economic status of family		
Poor	1518	40.61
Average	1926	51.52
Good	294	7.87
Years of schooling		
Below average (0 years)	1395	37.32
Average (1–6 years)	1183	31.65
Above average (7 years and above)	1160	31.03
Social cohesion		
Low(1–2 points)	1207	32.29
Fair (3–5 points)	1925	51.50
High(6–9 points)	606	16.21
Number of negative life events		
≥3	121	3.24
2	425	11.37
1	1213	32.45
0	1979	52.94
**Potential confounding variables**		
Gender		
Female	2242	59.98
Male	1496	40.02
Age		
70 year old and above	557	14.90
60–69	757	20.25
50–59	977	26.14
40–49	782	20.92
30–39	421	11.26
18–29	244	6.53
Marital status		
Never married	151	4.04
Divorced/separated/widowed	421	11.26
Married/remarried/cohabitating	3166	84.70
Self-perceived physical health		
Bad	1274	34.08
Fair	1479	39.57
Good	985	26.35
Ethnicity		
Han nationality	3720	99.52
Other ethnic minorities	18	0.48
Religion		
No	3551	95.00
Yes	187	5.00

Table 2 displays the distribution of variations in depression in the previous week according to years of schooling, self-reported economic status of the family, social cohesion in the previous year and negative life events at anytime in the past. Compared with above-average and average years of schooling, the prevalence of moderate and severe depression associated with below average years of schooling was clearly higher (5.34%, 8.88%, 13.19%, respectively), similar to the prevalence of moderate and severe depression and self-reported economic status (3.40%, 4.52%, 16.73% for good, fair and poor, respectively) and social cohesion (5.28%, 7.64%, 14.25% for high, fair and low, respectively). For negative life events, the prevalence of moderate and severe depression clearly increased with an increase in negative life events (67%, 11.05%, 30.82%, 43.80% with 0 events, 1 event, 2 events, and 3 events or more, respectively). In addition, x^2^ tests showed there were significant associations between variations in depression and variations in years of schooling, self-reported economic status, social cohesion and negative life events (*x^2^* = 69.70, *p<*0.0001; *x^2^* = 237.41, *p<*0.0001; *x^2^* = 56.44, *p<*0.0001; and *x^2^* = 723.56, *p<*0.0001, respectively).

**Table 2:** Cross-table analysis between Social Determinants of Health and depression (N = 3738).

Variables	No depression		Mild depression		Moderate and severe depression		*X* ^2^	*P*
	n	%	n	%	n	%		
**Self-reported economic status of family**								
Poor	1019	67.13	245	16.14	254	16.73	237.41	<0.0001
Average	1692	87.85	147	7.63	87	4.52		
Good	276	93.88	8	2.72	10	3.40		
**Years of schooling**								
Below average	1036	74.27	175	12.54	184	13.19	69.70	<0.0001
Average	938	79.29	140	11.83	105	8.88		
Above average	1013	87.33	85	7.33	62	5.34		
**Social cohesion**								
Low	889	73.65	146	12.10	172	14.25	56.44	<0.0001
Fair	1577	81.92	201	10.44	147	7.64		
High	521	85.97	53	8.75	32	5.28		
**Number of negative life events**								
≥3	41	33.88	27	22.31	53	43.80	723.56	<0.0001
2	205	48.24	89	20.94	131	30.82		
1	899	74.11	180	14.84	134	11.05		
0	1842	93.08	104	5.26	33	1.67		

Table 3 displays the rude ORs and 95% CIs for depression for all potential confounding variables and SDH variables. Results of the univariate logistic regression analysis suggested that all the potential confounding variables except gender were significantly associated with depression in the survey. First, after adjusting for significant potential confounding variables (including age, marital status and self-perceived physical health status), the associations between depression and self-reported family economic status, years of schooling, social cohesion and number of negative life events were still significant but weakened. However, compared with the group with high social cohesion, the group who had fair social cohesion was no more likely to experience mild, moderate or severe depression. Then, through stepwise logistical regression analysis, we obtained the final multivariable model including all significant variables. In the final model, all the SDH except years of schooling remained significant. Individuals who were divorced, separated, or widowed were more likely to experience mild, moderate or severe depression, compared with those who were married, remarried or cohabitating. Regarding self-perceived physical health status, participants with physical health self-reported as fair or poor were more likely to experience mild, moderate or severe depression, compared with the group with good self-perceived physical health.

**Table 3:** Unadjusted and adjusted associations between Social Determinants of Health and depression (N = 3738).

Variables	Crude OR (95%CI)	Adjusted OR (95%CI)^a^	Adjusted OR (95%CI)^b^
**The variables of Social Determinants of Health**			
**Self-reported economic status of family**			
Poor	7.50(4.62, 12.20)**	4.65(2.83, 7.66)**	3.60(2.17, 5.97)**
Average	2.09(1.127, 3.42)**	1.81(1.09, 3.00)**	1.76(1.06, 2.95)*
Good	1.00	1.00	1.00
**Years of schooling**			
Below average	2.42(1.96, 2.99)**	1.24(0.94, 1.63)	-
Average	1.79(1.43, 2.23)**	1.30(1.02, 1.66)*	-
Above average	1.00	1.00	-
**Social cohesion**			
Low	2.26(1.74, 2.94)**	1.66(1.25, 2.20)**	1.57(1.17, 2.11)**
Fair	1.36(1.05, 1.75)*	1.13(0.86, 1.48)	1.11(0.84, 1.48)
High	1.00	1.00	1.00
**Number of negative life events**			
≥3	28.49(19.55, 41.54)**	17.91(12.11, 26.47)**	15.15(10.22, 22.47)**
2	15.54(12.09, 19.98)**	10.01(7.70, 13.02)**	8.73(6.71, 11.37)**
1	4.75(3.83, 5.89)**	3.71(2.97, 4.63)**	3.57(2.85, 4.47)**
0	1.00	1.00	1.00
**Potential confounding variables**			
**Gender**			
Female	0.87(0.74, 1.03)		-
Male	1.00		-
**Age**			
70 year old and above	5.35(3.16, 9.05)**		
60–69	4.67(2.78, 7.84)**		
50–59	3.38(2.02, 5.68)**		
40–49	2.64(1.56, 4.48)**		
30–39	2.04(1.15, 3.60)*		
18–29	1.00		
**Marital status**			
Never married	1.33(0.90, 1.96)		1.49(0.95, 2.32)
Divorced/separated/widowed	2.29(1.85, 2.85)**		1.39(1.09, 1.79)*
Married/remarried/cohabitating	1.00		1.00
**Self-perceived physical health**			
Bad	10.06(7.58, 13.34)**		4.79(3.54, 6.46)**
Fair	2.25(1.66, 3.04)**		1.65(1.20, 2.26)**
Good	1.00		1.00

a Adjusting for significant control variables (including age group, marital status, self-rated physical health)

b Final multivariate model including all significant variables

*P<0.05;

** *P*<0.01 (two-tailed test);

OR, odds ratio; 95% CI, 95% Confidence Intervals

## Discussion

The cross-table analysis showed that variations in levels of depression were associated with variations in socioeconomic status, social cohesion and negative life events. Logistic regression analysis confirmed the associations even after adjusting for potential confounding variables. This finding supports our original hypothesis and provides evidence and clues regarding SDH and their implications for depression. Depression prevention and control should be related to social inequity. In particular, social inequity and the role of policy emphasized by SDH should be high priorities when addressing the issue of depression.

Previous studies have examined risk factors for depression, including social factors such as socioeconomic status, social support, religious practices and marital status [23], [30]–[31].The current study confirms existing research results. However, it is not enough to consider only social risk factors when addressing depression prevention and control. For example, considering socioeconomic status as a risk factor for depression suggests the need to improve the socioeconomic status of patients/individuals with depression. However, from the perspective of SDH, the low socioeconomic status of those with depression reflects social inequity, not individual socioeconomic status. Therefore, the guiding role of the SDH perspective is to promote social justice, not improve the socioeconomic status of patients/individuals. This perspective could provide valuable guidance related to interventions for depression, namely, changing depression from an individual issue to a public issue [7], [8], [14], [32]. To the best of our knowledge, there are few studies on the relationship of SDH and the prevention and treatment of depression.

The SDH perspective can enhance our understanding of variations in depression and the role of social factors. Depression should be of concern to all policy makers, not merely those within the health sector, so that intersectoral action can be taken. Furthermore, it is not an issue to be addressed only by psychiatrists. Previous studies of risk factors for depression have neglected to consider social inequity and the role of policy in addressing such inequity. To make further significant advances in depression research, a transdisciplinary approach is needed that integrates the study of the biological and social nature of depression, including the SDH. In addition, social theories research of mental health [33]–[35], including Durkheim's research, the Black Report and the Acheson Report, typically consider social determinants — what the WHO-CSDH refers to as the conditions in which people are born, grow, live and work — as the major determinants of mental health. The difficulty is that although the evidence linking the social world and health is strong, we are not entirely sure which aspects of the social, physical and economic environment influence mental health. We are also not sure about what policies can tackle this raft of interlinked problems. To accomplish this, following in the footsteps of the WHO-CSDH, the influence of SDH on depression should be further examined. In addition, policy research and applications should be taken seriously to promote mental health. This strategy represents a possible clue to theoretical innovations in prevention and treatment of depression as well as mental health.

Furthermore, according to the conceptual framework for action of the WHO-CSDH, the most important structural stratifiers and their proxy indicators include income, education, occupation, social class, gender and race/ethnicity. Not included is culture [8]. However, the effect of culture on depression and SDH is worth analysis and discussion [36]. Yeung et al. revealed that depressed Chinese Americans generally present with somatic symptoms [37]. Their study also supported findings from earlier studies that depressed Asian Americans rarely use mental health services [38]–[40]. They usually seek help from general hospitals instead. Similarly, in previous studies by Chen and Karasz et al. Asian Americans used emotion-descriptive terms for symptoms in vignettes, which was attributed to their reactions to social situations [41]–[43]. In mainland China, psychiatric patients tend to minimize emotional distress while emphasizing their physical suffering. They also rarely use mental health services and usually seek help from general hospitals instead, especially in rural China [40], [44], [45]. The existing studies consistently show that Chinese individuals, including migrants, want to neither report mental health-related symptoms nor seek mental health services. One important reason for this is that the Chinese culture, given its collectivistic nature, regards mental illness and even appearances at medical settings a stigma [46], [47]. Moreover, if such matters are talked about and thus known by others, individuals can be ridiculed and “lose face”. In fact, in China, low socio-economic status, low social recognition and low cultural status are considered stigmas, all of which essentially come from social inequality. The Chinese proverb, “In order to show oneself as a fat man (fat means wealthy in the Chinese culture), he makes his face swollen” is an example of low socio-economic status as a stigma in the Chinese culture. Therefore, it may be more appropriate for Chinese and Asian populations compared with the West to analyze and deal with depression issues from the perspective of SDH.

In China, much literature has emphasized the importance of mental health, especially with the social inequities and East–West conflict of values since the 1979 reform and opening-up policy [44], [45], [48], [49]. It is common that mental health services are a “one-man show” by psychiatrists. Those who provide mental health services are mostly limited to psychiatrists and psychiatric nurses, and the vast majority of them work only in psychiatric hospitals. The role of psychiatric nurses is limited to ward “guardian”. They have almost no therapeutic role besides dispensing pills to patients. At present, China has no psychiatric social workers and almost no clinical psychologists. As a result, community extended services and professional consulting services (including home visits) are almost non-existent [44], [45]. Combined with the above analysis and following in the footsteps of the WHO-CSDH, taking cell-to-society (seeing social inequality as the “causes of the causes” of SDH) and pill-to-policy (seeing policy action as social treatment targeted at social inequality) approaches should be encouraged in future research related to implications for depression.

There are a few limitations of the current study that may reduce the generalizability of our findings. First, the overall response rate was 59.58%, which may represent potential selection bias. Second, we used self-report measures to assess economic status and depressive symptoms. These are prone to participant response bias, such as low reported symptoms due to the stigmas mentioned above. Third, a cultural measure was lacking in the current study. In the era of economic globalization, it is important to research the cross-cultural generalizability of existing explanatory models of SDH in depression and draw respective conclusions for policy recommendations and action. Fourth, like all cross-sectional studies, it is difficult to establish causal association between independent and dependant variables. Future studies are needed to clarify these important issues.

Despite these limitations, the current study provides initial evidence of the importance of SDH in depression. Findings indicate that social inequity and the role of policy action emphasized by SDH should be considered high priorities when addressing the issue of depression. Furthermore, taking cell-to-society and pill-to-policy approaches should be encouraged in the future.
